# On the Importance of Characterizing Virtual PMUs for Hardware-in-the-Loop and Digital Twin Applications

**DOI:** 10.3390/s21186133

**Published:** 2021-09-13

**Authors:** Alessandro Mingotti, Federica Costa, Diego Cavaliere, Lorenzo Peretto, Roberto Tinarelli

**Affiliations:** Department of Electrical, Electronic and Information Engineering, Guglielmo Marconi Alma Mater Studiorum, University of Bologna, Viale del Risorgimento 2, 40136 Bologna, Italy; federica.costa13@unibo.it (F.C.); diego.cavaliere2@unibo.it (D.C.); lorenzo.peretto@unibo.it (L.P.); roberto.tinarelli3@unibo.it (R.T.)

**Keywords:** hardware-in-the-loop, phasor measurement unit, digital twin, calibrator, characterization, sensors, real-time simulator, distribution network

## Abstract

In recent years, the introduction of real-time simulators (RTS) has changed the way of researching the power network. In particular, researchers and system operators (SOs) are now capable of simulating the complete network and of making it interact with the real world thanks to the hardware-in-the-loop (HIL) and digital twin (DT) concepts. Such tools create infinite scenarios in which the network can be tested and virtually monitored to, for example, predict and avoid faults or energy shortages. Furthermore, the real-time monitoring of the network allows estimating the status of the electrical assets and consequently undertake their predictive maintenance. The success of the HIL and DT application relies on the fact that the simulated network elements (cables, generation, accessories, converters, etc.) are correctly modeled and characterized. This is particularly true if the RTS acquisition capabilities are used to enable the HIL and the DT. To this purpose, this work aims at emphasizing the role of a preliminary characterization of the virtual elements inside the RTS system, experimentally verifying how the overall performance is significantly affected by them. To this purpose, a virtual phasor measurement unit (PMU) is tested and characterized to understand its uncertainty contribution. To achieve that, firstly, the characterization of a virtual PMU calibrator is described. Afterward, the virtual PMU calibration is performed, and the results clearly highlight its key role in the overall uncertainty. It is then possible to conclude that the characterization of the virtual elements, or models, inside RTS systems (omitted most of the time) is fundamental to avoid wrong results. The same concepts can be extended to all those fields that exploit HIL and DT capabilities.

## 1. Introduction

The normal and correct operation of the power network is achievable and can be maintained only with proper monitoring infrastructure. Such infrastructure mainly depends on (i) the economic availability of the system operator (SO); (ii) the portion of the considered network, which could be the transmission network (TN) or the distribution network (DN); (iii) the quantities to be measured and the desired target accuracy. Adding more details, the topology of the TN facilitates the installation and spread of distributed monitoring systems (DMSs). In fact, long lines and a few-nodes structure are easier to monitor than the meshed and crowded nodes structure of the DN [1,2,3]. As for the target uncertainty, it is fundamental, during the design and development phase of a DMS, to fix it in order to establish the minimum requirements that the monitoring devices must have. This is a critical choice because the final cost of the single measurement unit will significantly differ (even several orders of magnitude) depending on it.

A typical DMS consists of three main components: (i) the sensing element, (ii) the acquisition system, and (iii) the data storage. The latter element may be included in the acquisition system; however, cloud-based storage systems are becoming more popular and adopted; hence, they are worthy of being mentioned.

The main sensing elements are the instrument transformers (ITs), which measure voltages and currents, scaling them to suitable values for the acquisition systems. Of course, environmental quantities can be measured as well (such as in [4,5,6,7] for cables, and in [8,9,10] for insulators, etc.) but out of the scope of this paper. Focusing on the distribution side of the network, ITs must be reliable in all the operating conditions of the grid, which may include (i) temperature and environmental conditions variations, (ii) low power quality (PQ), (iii) off-nominal values of voltages and currents, etc. To this purpose, the IEC 61869 standard series regulates most of the aspects associated with ITs. In particular, IEC 61869-1 and -6 [11,12] provide general requirements on legacy inductive ITs and on low-power instrument transformers (LPITs), respectively. All the other documents of the series treat specific types of ITs such as current and voltage inductive transformers (CTs and VTs), electronic ITs (EITs), etc. In parallel, researchers are very active and prolific regarding the studies on ITs. For example, the modeling of ITs and LPITs is described in [13,14,15,16]. In detail, [13] treats voltage transformers, [14] non-conventional ITS, and [15,16] tackles current transformers.

The characterization and the accuracy aspects related to the operation of inductive and non-conventional ITs are discussed in [17,18,19,20,21,22,23]. In particular, [17] describes the calibration of traditional and electronic transformers, while [18,21,22] are specific for current transformers. Finally, performance assessment when ITs are working at off-nominal conditions (including high frequency, temperature, etc.) and new methods to compensate for such problems are presented in [24,25,26,27,28,29]. For example, [27] characterizes ITs for power quality applications, while [29] uses datasets of fault waveforms for the current transformer testing.

In recent years, the above studies have been supported by new tools for network monitoring: real-time simulators (RTS). Such tools allow SOs and researchers to simulate and run portions or complete networks with the aim of better understanding them and predicting their behavior. In particular, RTSs require models for the assets and elements of the grid, which consider nominal and off-nominal operations. Furthermore, the simulating environment needs accurate and realistic generation and load profiles to avoid incoherent and wrong results.

**Figure 1:**
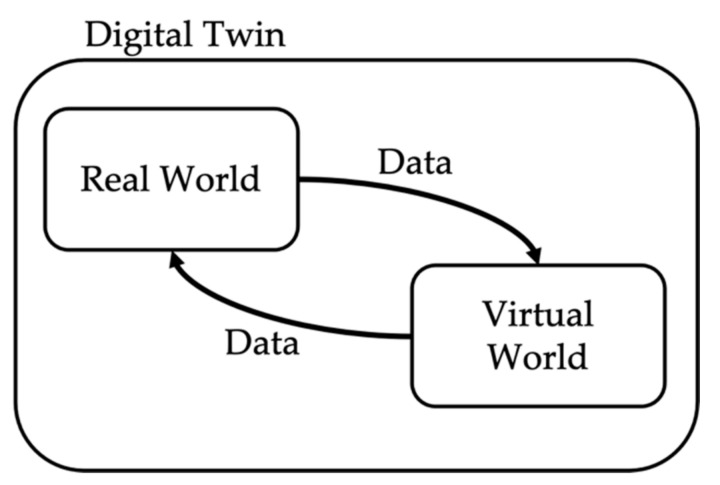
Schematic representation of the DT concept.

RTS systems enable different types of simulations. Among them, two promising techniques are hardware-in-the-loop (HIL) and the digital twin (DT). The former technique consists of exploiting the analog/digital input/outputs of the RTS to include real devices inside the simulation [30,31,32,33,34]. This way, the information from the physical world can be included in the virtual environment. As for the DT, instead, it enhances the previous concept, upgrading it to the level at which the information obtained in the virtual world is used to improve the physical one, and vice versa [35,36,37,38]. In other words, there is a real-time interaction and mutual information exchange between the virtual and physical worlds. See Figure 1 for a schematic representation of the DT concept. Both techniques are extremely powerful, and they have been used in various fields (e.g., agriculture, manufacturing, modeling, etc.).

Note that, as it has been described for ITs, RTS systems must also be characterized and reliable enough according to the target uncertainty. On the contrary, the risk is to include in the measurement system or in the DT environment an element in which characteristics are far worse than those of the other devices.

To this purpose, and considering that such an aspect is not always considered or sufficiently treated, this article aims at emphasizing the importance of treating the RTS exactly as the other devices. In fact, RTSs and virtual models of physical devices should be characterized and assessed as well in order to avoid unexpected sources to the overall uncertainty. Therefore, this work has a double added value. First, a calibrator for RTS systems is presented and characterized. Second, the described calibrator is used to characterize a virtual phasor measurement unit (PMU) developed inside an RTS (the OPAL). Note that this study focuses on PMUs and the electrical world; however, the main concept can be extended and implemented in all fields in which simulations and DT are being used.

Of course, the literature already includes several works on virtual PMUs and RTS for HIL and DT applications. For example, [39] described a PMU-RTS HIL test bed that aims at characterizing physical PMUs. In [40,41,42], virtual PMUs have been developed and implemented inside a DT environment. Complete DT infrastructure consisting of physical PMUs and RTS-based simulation systems has been described in [43,44,45]. Finally, the impact of time delays inside HIL application is studied in [46].

However, the added value of this work consists of the specific analysis of the contribution to the uncertainty of the RTS system and, in particular, of its virtual elements, which are typically neglected or not depending on when the simulations are performed. Furthermore, the characterization procedure for a calibrator, aimed at assessing the performance of the RTS and its virtual elements, is described in detail.

The following sections are structured as follows. Section 2 introduces the selected calibrator and describes the entire characterization process. The adopted RTS is presented in Section 3 together with the virtual PMU. Section 4, the core section, contains the characterization of the virtual PMU and the uncertainty propagation process aimed at quantifying the characterization accuracy. Finally, the summary of the work and a conclusion are given in Section 5.

## 2. Characterization of the Calibrator

As mentioned in the previous section, the first operation to be accomplished is the characterization of the calibrator, which is presented herein. The final goal is to assess the uncertainty that can be reasonably associated with the reference test waveform generated by the calibrator. Once this operation has been performed, the virtual PMU performance can finally be evaluated. The present article only deals with the steady-state characterization for the M-class PMU because the P-class has less strict requirements, hence it is included in the M-class. The virtual PMU, in accordance with its standard IEC 60255-118-1 [47], has a reporting rate of 1 frame per second (fps); consequently, the dynamic performance requirements and the out-of-band requirements do not apply.

The remainder of this section contains: first, the calibrator concept and hardware are presented in Section 2.1; second, the designed characterization tests for the calibrator are described in Section 2.2; finally, the characterization results are commented on and arranged to provide the accuracy specifications of the calibrator in Section 2.3.

### 2.1. The Calibrator Hardware Architecture

The standard [47] prescribes the verification of some parameters that quantify the deviation of the synchrophasor measured by the tested PMU from the reference one. These parameters are the Total Vector Error (TVE), the Frequency Error (FE), and the Rate Of Change Of Frequency (ROCOF) Error (RFE). The uncertainty affecting the reference synchrophasor should be at least one order of magnitude smaller than the one expected from the tested PMU. To conduct this kind of evaluation, a PMU test system (or PMU calibrator) is needed. In recent years, many have faced the problem of PMU calibration. Besides the research on the definition of accurate phasor estimation algorithms [48,49,50,51], the implementation of reference class hardware test systems is of key importance to the successful deployment of PMUs in Smart Grids. Researchers and national metrological institutes have developed in-house test facilities [52,53,54,55,56,57,58,59] and employed off-the-shelf solutions [60,61,62]. The architecture of PMU calibrators is quite consolidated. Basically, the PMU calibrator generates the test waveforms through an analog output stage and feeds them to the PMU under test; simultaneously, the calibrator returns the generated waveform to produce the reference synchrophasor. Both the generation and the acquisition stages are driven by a timing stage distinguished by stable clocks and triggers referenced to an absolute timing source, such as a GPS clock or an atomic clock. The main reason behind this kind of design, in which a reference PMU is actually implemented, is the fact that it also allows the calibration of other calibrators [55].

**Figure 2:**
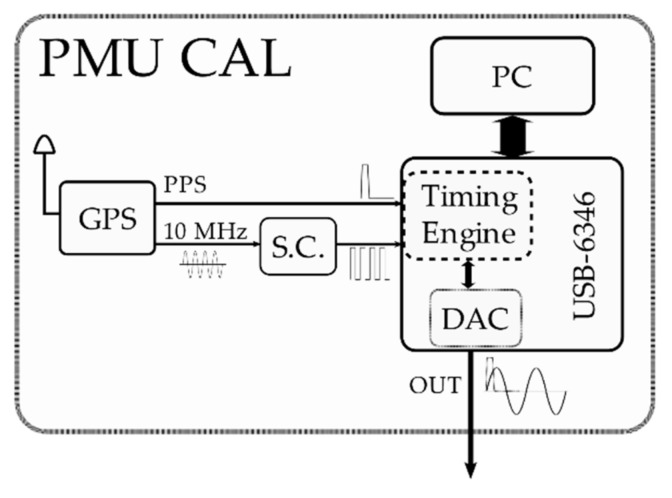
Schematics of the calibrator architecture.

For the purpose of the present work, this aspect is not necessary: thus, the calibrator architecture has been kept as simple as possible. In fact, it is a generator capable of producing an accurate waveform from the magnitude and timing point of view, equivalent to the reference synchrophasor. The components are sketched in Figure 2: (i) an accurate GPS disciplined oscillator Trimble Thunderbolt E [63] providing the pulse-per second (PPS) signal and a disciplined 10 MHz reference clock signal; (ii) an NI USB-6346 multifunction I/O device [64], which employs as timing and synchronization sources both the PPS and the 10 MHz signals and outputs the test waveforms from the analog output channel; (iii) a PC running the calibrator software and the calibrator characterization test software, both developed in LabVIEW. The characteristics of the analog output channel (OUT) DAC are summarized in Table 1, whereas the characteristics of the GPS disciplined oscillator are shown in Table 2.

The block denoted as S.C. is an active electronic circuit that only adapts the 10 MHz AC sinusoidal reference signal to a 10 MHz TTL-compatible digital signal.

The internal timing engine of the USB-6346 board is equipped with a phase-locked loop (PLL), which allows deriving the device’s main 100 MHz time base from the external 10 MHz reference clock. This means that the DAC sample clock is consequently derived from the external reference clock. The PPS signal serves as a trigger for starting the DAC operations.

### 2.2. Characterization and Testing of the Calibrator

In this sub-section, the adopted test procedures and setups are shown and explained. To characterize the steady-state test waveforms to be applied to the PMU under test, it is necessary to address the magnitude, the phase angle, and the frequency uncertainties. Note that the calibrator hardware is also affected by the timing and the synchronization non-idealities: as defined in [52], the timing non-idealities are the ones related to the deviations from the UTC second rollover, while the synchronization non-idealities are due to the latencies and jitters in the calibrator hardware. These phenomena have direct consequences on the resulting reference waveform phase.

In what follows, if not stated otherwise, the test waveform reproduced by the calibrator is a zero-phase, 50 Hz, sinusoidal waveform with a magnitude equal to 5 V (corresponding to 100% of the rated value for the OPAL RTS analog input, as explained in sub-Section 2.2.1). The DAC sampling rate for all the tests discussed in this article is 800 kSa/s.

#### 2.2.1. Signal Magnitude Test Case

The test waveform magnitude characterization has been considered first. The calibrator analog output is connected directly to the input of a digital multimeter (DMM) HP 3458A. The DMM is set in synchronous sampling mode, which is the most accurate configuration for AC voltage measurement [65]. The standard [47] requires that the PMU shall be tested with:

- A voltage signal varying from 80 to 120% of the rated value;
- A current signal varying from 10 to 200% of the rated value.

In a HIL PMU test scenario, the virtual PMU would interact with an electrical power source (voltage and current) by means of proper ITs and signal conditioning stages in between to match the OPAL A/D converter’s full dynamic range (±10 V). Consequently, a successful design would imply the usage of the full A/D range when the current is 200% of the rated value. Given these considerations, the calibrator has been characterized in correspondence with the test points collected in Table 3.

For each test point, 50 repetitions have been performed. Finally, the values measured by the DMM have been compared against the magnitudes set on the calibrator user interface.

#### 2.2.2. Harmonic Distortion Test Case

To prove the synchrophasor measurement consistency, the PMU under test should be evaluated even in the presence of harmonic disturbances added to the main power frequency signal. The harmonic disturbance is defined as a single harmonic, up to the fiftieth order, with a magnitude equal to 10% of the power-frequency-signal rated value. Therefore, the calibrator capability of generating this reference waveform has been experimentally checked, connecting its output again to the DMM. It shall be considered that the HP 3458A basic accuracy specifications strictly apply to sinusoidal signals. In [65], an additional “crest-factor error” term can be found. It is well known that the crest-factor error information without any additional indications on the bandwidth is not sufficient to properly evaluate the DMM performance. However, some observations can be made: (i) the harmonic components’ amplitudes determine a worst-case crest factor of ≈1.5, resulting in an additional reading error of 0.001%; (ii) the harmonic components’ frequencies are only a few times bigger than the fundamental frequency, and they are contained by far in the bandwidth in which the DMM presents good overall accuracy. In such a scenario, it could be reasonably stated that the DMM accuracy can still be reliably assessed from its specifications. Nevertheless, first, the calibrator has been set to generate only the single harmonic components; second, the test waveform containing both the 50 Hz component and the harmonic component has been produced. A comparison between the single harmonic signal and the standard harmonic disturbance test waveform has been performed. The test cases are summarized in Table 4.

For each test point, 50 repetitions have been performed. Finally, the values measured by the DMM have been compared against the magnitudes set on the calibrator user interface.

#### 2.2.3. Synchronization Test Case

The synchrophasor measurements are a time-critical application: a timing error of only 31.7 µs causes a 1% TVE error when there is no magnitude error. Thus, it is very important to analyze the behavior of the calibrator from this point of view.

**Figure 3:**
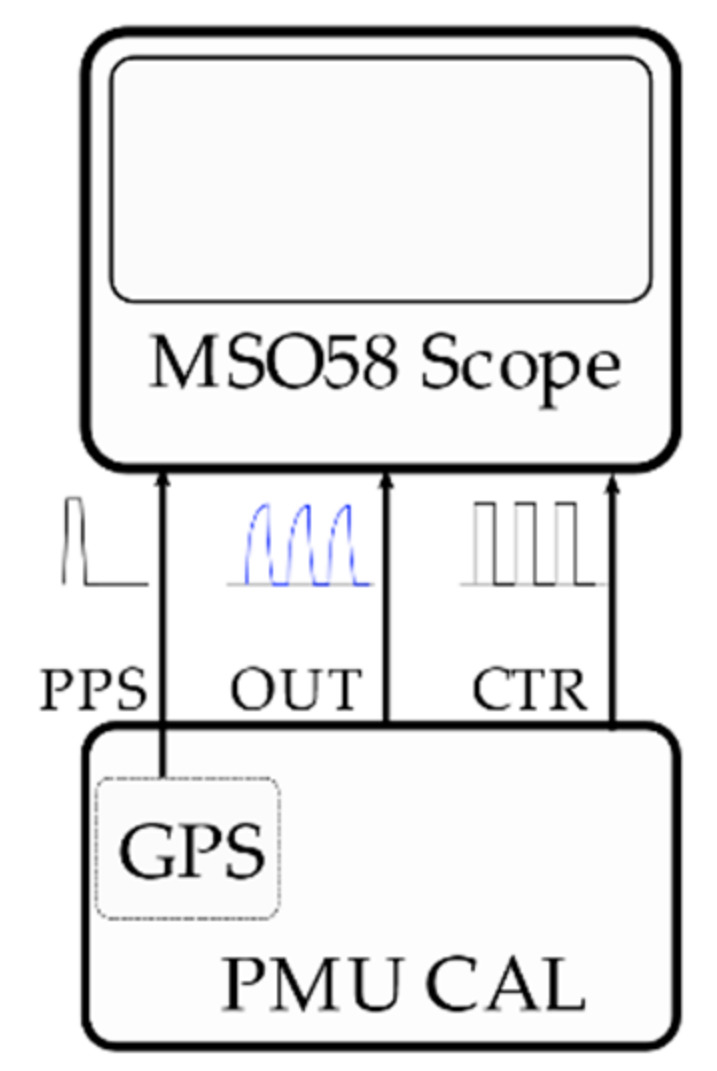
Schematics of the calibrator synchronization test.

The synchronization uncertainty has been evaluated by means of a Tektronix MSO58 12 bits, 3.125 GSa/s oscilloscope [66], as shown in Figure 3. First, the PPS signal from the GPS disciplined clock is also sent to the oscilloscope; second, the USB-6346 has been programmed to also output a square wave from the on-board counter sub-system (CTR); finally, the DAC outputs a square wave (OUT) with the same parameters (amplitude, frequency, and duty-cycle) of the one generated by the counter. This setup allows the evaluation of the calibrator response delay and jitter compared to the PPS signal. Two different square wave frequencies have been examined: 50 Hz and 100 kHz. For each case, 1000 acquisitions have been taken.

Concerning the timing uncertainty, only the GPS disciplined oscillator uncertainty is considered; in fact, the GPS time has been chosen as a UTC time reference. Since the OPAL RTS receives the same PPS signal as the calibrator, there are no additional contributions due to the presence of different timing sources. For instance, if the OPAL RTS and the calibrator had been equipped with different GPS receivers, then the two PPS signals would have been slightly misaligned between each other, even though they would have been under the same sky conditions.

#### 2.2.4. Frequency and ROCOF Test

The frequency uncertainty has been checked by means of the frequency counter built in the Rigol DG 1022Z arbitrary waveform generator, which provides an accuracy of 1 ppm. The calibrator’s test sinusoidal waveform frequency has been tested over the range from 45 to 55 Hz, in steps of 0.5 Hz. For each frequency test point, 100 measurements have been taken. The values measured by the frequency counter have been compared against the frequency value set on the calibrator user interface. Finally, the frequency counter readings have also been used to estimate the calibrator ROCOF since it is basically defined as a variation of the frequency between sequential measurements.

#### 2.2.5. Phase Displacement Test

**Figure 4:**
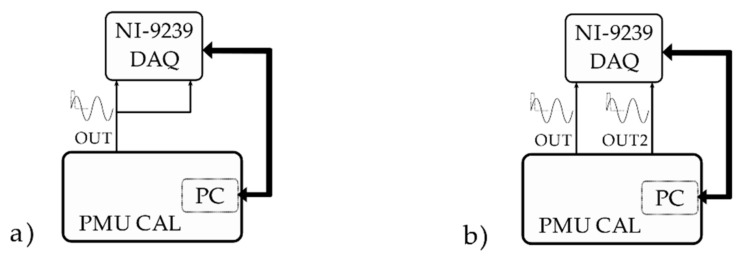
Schematics of the calibrator phase displacement test: (**a**) “actual zero” test setup; (**b**) 0, 30, 45, 90° phase-shifted sinusoids test setup.

An additional investigation has been performed to estimate the calibrator’s accuracy in the matter of reproducing the desired initial phase of the sinusoidal test waveform. The test setups are reproduced in Figure 4. To achieve this, the calibrator synthetizes a sinusoidal waveform from each of its two analog output channels (OUT and OUT2), both triggered simultaneously by the PPS. The initial phase of the second sinusoid has been kept fixed and equal to 0 (OUT2), while the initial phase of the first sinusoid has been progressively increased (OUT). In particular, four phase displacements have been tested: 0°, 30°, 45°, 90°. A NI-9239 data acquisition board (DAQ) has been used to simultaneously acquire the two generated waveforms. The phase displacement has been measured in the frequency domain, and a 10 s long time record has been acquired on each channel to mitigate spectral leakage impairments. Furthermore, a verification of the “actual zero phase displacement” seen by the DAQ channels has been conducted, too, by feeding the same signal to both. A total of 100 repetitions have been carried out for each case. Note that the NI-9239 operation is not triggered by the PPS signal, but this does not matter since the measured quantity is a difference between two steady-state waveforms.

Finally, the phase displacement values have been compared against the phase displacement set on the calibrator user interface.

### 2.3. Results of the Characterization Tests

This section presents the results obtained from each of the test cases described above.

#### 2.3.1. Signal Magnitude Test Results

In Table 5, the following quantities are reported: $X_{1}{\;\mathrm{RMS}}^{*}$ is the ideal RMS value of the sinusoidal test waveform set on the calibrator user interface corresponding to the test points shown in Table 3; $\mu_{X_{1}\;\mathrm{RMS}}$ is the average of the RMS values measured by the DMM and $\sigma_{X_{1}\;\mathrm{RMS}}$ is the standard deviation; $u_{{A,\;X}_{1}\;\mathrm{RMS}}$ and $u_{{B,\;X}_{1}\;\mathrm{RMS}}$ are the uncertainties evaluated according to Type A and Type B methods, respectively, as described in [67]. Lastly, $\Delta_{X_{1}\;\mathrm{RMS}}$ is a parameter defined as:

(1) $$\Delta_{X_{1}\;\mathrm{RMS}}{=X}_{1}{\;\mathrm{RMS}}^{*}-{\;\mu }_{X_{1}\;\mathrm{RMS}}$$

The main contribution to the uncertainty comes from the DMM a priori evaluation of the measurement uncertainty; in fact, the deviation in the measurement is negligible compared to the former. Moreover, the computed values of $\Delta_{X_{1}\;\mathrm{RMS}}$ show that the deviation between the RMS value of the sinusoidal test waveform produced by the calibrator and the ideal RMS value set on the calibrator user interface is of the same order of magnitude of the uncertainty affecting the DMM measurement.

#### 2.3.2. Harmonic Distortion Test Results

Given the considerable amount of test points, for readability’s sake, only the single harmonic cases which produced the best and the worst results have been reported in Table 6. The quantities other than the harmonic order h are the same as the ones in Table 3, but this time they refer to the tested single harmonic component.

The order of magnitude of the $\Delta_{X_{h}\;\mathrm{RMS}}$ values for all the others h cases is $10^{-5}$. Not surprisingly, considerations analogous to the ones deduced in Section 2.3.1 can be made. In fact, as in the previous case, the DMM acquires sinusoidal signals whose frequency is contained in a bandwidth in which the DMM maintains almost the same accuracy.

Table 7 is analogous to Table 6, but the values for the test signal composed by the power frequency component and the harmonic disturbance are reported.

Again, the results obtained are in line with the previous ones, confirming the consistency of the operations.

#### 2.3.3. Synchronization Test Results

**Figure 5:**
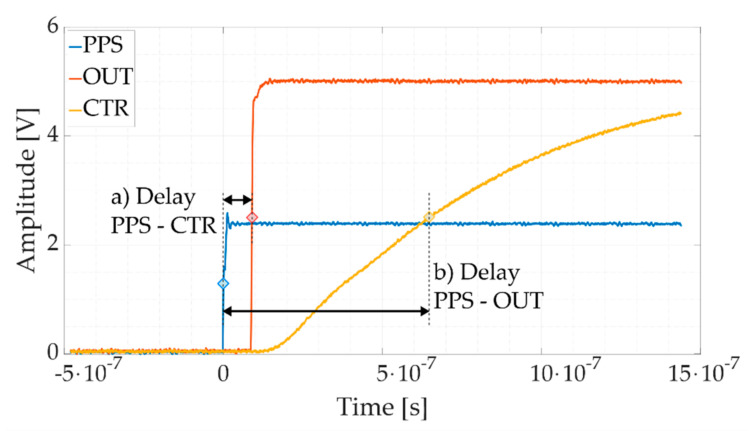
Oscilloscope waveform acquisitions for the board synchronization evaluation. The PPS signal (**blue**), the CTR signal (**red**), the OUT signal (**yellow**).

**Figure 6:**
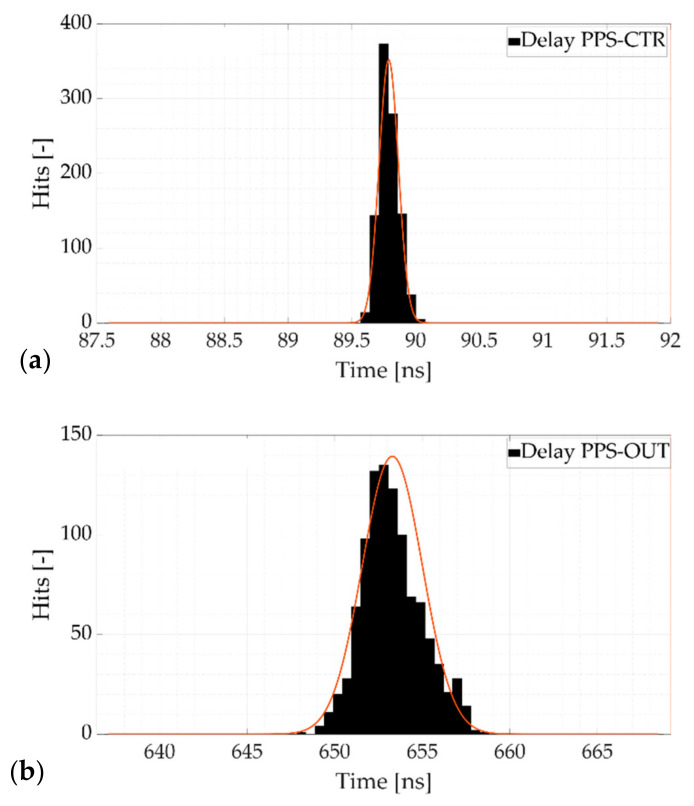
Distribution histograms of the delay measurement between the (**a**) PPS and the CTR rising fronts and (**b**) the PPS and the OUT rising fronts.

In Figure 5, the PPS signal, the 100 kHz square waveform generated from the analog output (OUT) of the calibrator and the digital square wave reproduced by the digital counter (CTR) are plotted. Figure 6, instead, shows the histograms representing the distribution of the delay measurement between the (a) PPS and the CTR rising fronts and (b) the PPS and the OUT rising fronts.

Other than the locked phase relation among the three waveforms, it is evident that the board outputs the requested signals in a very responsive way. The CTR signal rises almost a single time-base clock tick (≈90 ns) after the detection of the trigger, with low dispersion (less than 100 ps), denoting an immediate response of the board. The OUT signal response behavior shows a systematic delay contribution of ≈ 650 ns and a standard deviation of less than 2 ns (equivalent to ≈0.6 μrad).

The results for the case of the 50-Hz square waveforms are not reported since they do not differ significantly from the ones already shown.

#### 2.3.4. Frequency and ROCOF Test Results

Table 8 collects the measurements from the frequency characterization of the calibrator. The quantities are: $f^{*}$ is the frequency reference value set on the calibrator user interface; $\mu_{f}$ is the average frequency measured by the counter; $u_{A,\;f}$ and $u_{B,\;f}$ are the corresponding uncertainties evaluated according to Type A and Type B methods, respectively; finally, $\Delta_{f}$ is an error parameter defined as:

(2) $$\Delta_{f}{=f}^{*}-{\;\mu }_{f}.$$

Moreover, two parameters that could approximately quantify the ROCOF of the calibrator are represented by $\mu_{\mathrm{ROCOF}}$ and $\delta_{\max-\min}$.

The former is defined as:

(3) $$\mu_{\mathrm{ROCOF}}=\frac{1}{N-1}\sum_{i=1}^{N-1}f_{i+1}-{\;f}_{i},$$

where N=100 is the number of readings taken by the frequency counter for each value of $f^{*}$ and $f_{i}$ is the *i-th* frequency counter readings. $u_{A,\;\mathrm{ROCOF}}$ and $u_{B,\;\mathrm{ROCOF}}$ are the associated uncertainty evaluated according to the Type A and Type B method, respectively. In particular, the latter is evaluated as

(4) $$u_{B,\;\mathrm{ROCOF}}=\sqrt{{2u}_{B,f}}$$

since it is a difference between measured frequency values. The parameter $\delta_{\max-\min}$ is simply the difference between the maximum and the minimum values recorded by the counter at each $f^{*}$.

The presented results show that the measurement repeatability is less than the frequency counter a priori evaluation of the measurement uncertainty. Additionally, the deviation $\Delta_{f}$ is under that threshold. Even the ROCOF-related quantities suffer the frequency accuracy since they are computed from the measurement uncertainty readings. A final consideration on the evaluation of the ROCOF: since this parameter is the difference between the frequency values at two consecutive reporting instants, in a steady-state scenario, it should ideally be equal to zero. If the waveform synthetized by the calibrator is close to the ideal one, then it is reasonable to expect that its frequency would be almost constant, showing a very small deviation over the entire time interval during which it is examined, not only between two consecutive measurements separated by the reporting interval. In other words, if the reference generator can reproduce a sinusoidal waveform distinguished by a stable frequency over the testing time, then the ROCOF will be small. Hence, this means that an assessment of the stability (expressed in Hz) through the parameters $\delta_{\max-\min}$, $\mu_{\mathrm{ROCOF}}$ and $u_{A,\;\mathrm{ROCOF}}$ can also be used for studying the ROCOF (expressed in Hz/s).

#### 2.3.5. Phase Displacement Test Results

First, the results obtained from the “actual zero phase displacement” test are shown in Table 9.

Where $\mu_{\phi 0}$ is the average phase displacement between the two DAQ channels when they are sampling the same sinusoidal signal generated by the first analog output channel (OUT) of the calibrator, whereas $u_{A,\;\phi 0}$ is its associated standard uncertainty evaluated by means of the Type A method. This measurement shows the threshold of what can be considered as null phase displacement.

Then, the results obtained from the tested four phase displacements are shown in Table 10.

The quantities $\mu_{\phi }$ and $u_{A,\;\phi }$ are the same quantities as $\mu_{\phi 0}$ and $u_{A,\;\phi 0}$, but this time they are related to the measurement of the phase displacement between the sinusoidal waveforms generated by the calibrator’s two analog output channels, OUT and OUT2. The angles $\phi_{\mathrm{OUT}}^{*}$ and $\phi_{\mathrm{OUT}2}^{*}$ are the values of the synthetized sinusoids’ initial phases set in the calibrator user interface. Finally, $\Delta_{\phi }$ is:

(5) $$\Delta_{\phi }=(\phi_{\mathrm{OUT}}^{*}-\phi_{\mathrm{OUT}2}^{*})\;-{\;\mu }_{\phi }$$

Looking at Table 9 and Table 10, the first noticeable thing is the difference between $\mu_{\phi }$ and $\mu_{\phi 0}$ other than their relatively small dispersion, which underlines an actual difference between the two scenarios, probably because the two calibrator analog outputs are not identical to each other. Shifting the focus on Table 10, the value $\Delta_{\phi }$ is substantially the same for all the four phase displacement cases, suggesting the presence of a phase offset between the output channels.

#### 2.3.6. Characterization Conclusions

After the presentation of the calibrator characterization results in this sub-section, it is possible to summarize them with the goal of drawing an overall picture of the device’s performances.

Concerning magnitude, the calibrator has proved to be very accurate and precise in terms of reproducing waveforms with the desired RMS value. In the sinusoidal steady-state case, there are no appreciable deviations between the DMM measurement result and the ideal value set on the calibrator user interface. Moreover, the worst relative uncertainty is $1\;\times 10^{-4}$. The results are also similar in the harmonic test cases. This fact ensures us that the calibrator can provide the virtual PMU with the designed harmonic test waveform.

Analogously, the calibrator performs well also under the frequency point of view. No significant deviation has been observed from the frequency counter measurement results, and the worst relative standard uncertainty is ${6\;\times \;10}^{-7}$. Instead, the ROCOF absolute standard uncertainty is ${4\;\times \;10}^{-5}$ Hz.

Instead, different main contributions shall be considered for the phase accuracy. First, the 15 ns (≈5 μrad) introduced by the GPS disciplined oscillator; second, the 2 ns deviation (≈0.7 μrad) measured between the rising front of the PPS signal and the analog output step waveform; third, the 0.4 μrad deviation measured with the DAQ; finally, since errors on frequency directly affect the phase, it is possible to also add a contribution which translates the worst frequency uncertainty in an angle, resulting in a ≈6 μrad term. All the conversions from time and frequency to angles have been carried out considering the most precautionary scenario: for example, a 1 ns variation at 55 Hz corresponds to a bigger phase angle rather than the one at 45 Hz, whereas a 10 μHz variation corresponds to a bigger phase angle variation at 45 Hz rather than at 55 Hz. Combining all these components as the root of the sum of the squares and considering a 3σ interval, the phase uncertainty is equivalent to $\approx {2\;\times \;10}^{-5}\;\mathrm{rad}$.

Let us take under examination the equation below shown in [47]:

(6) $$\mathrm{TVE}=\sqrt{2\left(1+\mathrm{ME}\right)\left(1\;-\cos\left(\mathrm{PE}\right)\right){+\mathrm{ME}}^{2}}$$

where ME is the synchrophasor magnitude error and PE is the synchrophasor phase error. Given the conclusions of the analysis presented above, if ME = ${1\;\times \;10}^{-4}$ and PE = ${2\;\times \;10}^{-5}\;\mathrm{rad}$, then the equivalent TVE of the calibrator is approximately ≈0.01 %. This result is compliant with what is recommended in [47] for PMU test systems.

## 3. RTS Environment

This section aims at briefly presenting the RTS environment selected for being tested. In particular, the main features of the OPAL simulator are summarized in Section 3.1, while in Section 3.2, the virtual PMU to be tested is presented and commented on.

### 3.1. Description of the RTS

**Figure 7:**
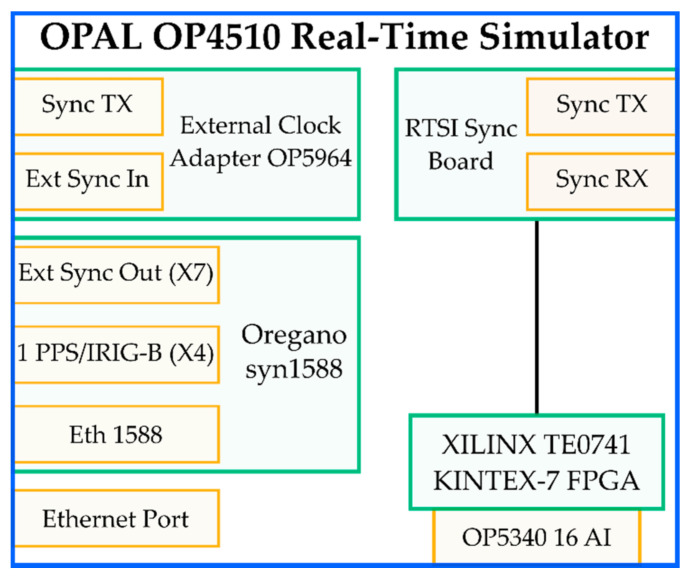
Main components of the OPAL OP4510 RTS.

The RTS adopted in this work is the OPAL-RT OP 4510 Simulator, which allows running real-time simulations and HIL applications via RT-LAB software. Its main components are depicted in Figure 7, in which each color has a specific meaning. Green is used for the internal components and yellow for the interfaces and connections.

A brief description of the main components is given in what follows:

- Oregano syn1588 PCIe NIC. It is a PCI Express Ethernet network interface that provides highly accurate clock synchronization via the IEEE 1588 Standard (accuracy of its oscillator higher than 0.05 ppm). The Oregano card can be synchronized with either a PPS signal or an IRIG-B signal from a GPS source (3.3 V signal).
- External Clock Adapter OP5964. It is used to receive and transmit the synchronization signal from the outside to the interfaces.
- RTSI (Real-Time System Interface) Synchronization Board. This board directly communicated with the FPGA, as can be seen from Figure 7 (black line).
- XILINX TE0741 KINTEX-7 FPGA. It accepts either OPAL-RT boards or RS422 signals. The types of synchronization allowed are LVDS and fiber optic.
- Analog input (AI) card OP5340. It features 16 synchronous differential analog input channels with a maximum voltage range of ±20 V, sampled at 400 kSa/s. The analog to digital converter (ADC) has a 16-bit resolution, and the minimum acquisition time is 2.5 µs per channel. The declared maximum noise of the analog card is 20 mV peak-to-peak. The ADC already includes anti-aliasing filters to remove frequencies higher than 600 kHz.
- Ethernet port. It is used to interface a laptop to the OP4510 RTS.

### 3.2. Description of the PMU

A PMU is a digital device that provides synchronized voltage and current phasor measurements referred to as synchrophasors. At the installation bus, instrument transformers, such as CTs and VTs, are needed to measure the three-phase quantities. Their analog signals are converted into digital by means of an ADC with a sampling rate usually varying from 12 to 128 samples per cycle of the rated power frequency.

In a PMU, the sampling clock could be phase-locked with a single time reference, which is given by the GPS pulse per second (PPS). The clock provided by the GPS system is also referred to as Universal Time Coordinated (UTC), and it is used as a time reference to time-tag the outputs.

At this stage, the phasor must be retrieved, and the easiest method to perform this action consists of using the Discrete Fourier Transform (DFT), which allows obtaining the magnitude and phase of the signal. Nevertheless, it must be highlighted that the relevant international standards, such as IEC 60255 [47], do not provide a specific phasor algorithm to be implemented in PMUs. Likewise, the window length, the sampling rate, the phasor estimates reporting rate, the communication protocol, as well as the measurement accuracy are all distinctive to each PMU device. Therefore, alternatives to the DFT technique have been investigated and reported in the scientific literature.

Two main algorithm categories can be distinguished: DFT-based and non-DFT-based algorithms. Examining the first category, the classic DFT-based methods work well when the system frequency is close to the nominal frequency but, due to spectral leakage, significant errors arise when the frequency drifts from its rated value. For this reason, much effort has been made in order to improve the accuracy of DFT-based estimation algorithms under off-nominal frequency conditions. Among these, it is worth mentioning interpolated-DFT approaches (IpDFT) [68,69] and dynamic DFT ones [70].

On the other hand, the majority of non-DFT-based algorithms are based on Kalman filters (KFs). It is worth remarking on Taylor-Kalman Filters (TKFs) [71] and Adaptive and Extended Kalman Filters (AKFs, EKFs) [72,73]. A small number of other approaches are based on different techniques such as Taylor Weighted Least-Squares (TWLS) [74], Space Vectors (SVs) [75], and wavelets [76]. Given the broadness of the topic enveloping several techniques, comparative studies between PMU estimation algorithms have also been presented in the literature.

Assessments between DFT and KF-based algorithms are typically performed, as in [77,78]. The analyses are based on simulations in accordance with [77] to evaluate and compare the performance of the estimators. It is shown that KFs are optimal for harmonic rejection and for tracking large-frequency deviations occurring in power systems, contrary to DFT-based ones, which suffer from leakage issues as aforementioned [77]. On the contrary, DFT approaches do not suffer from instabilities, in contrast to KFs, and they are generally simpler than the latter, resulting in a significantly reduced computational burden.

For this reason, in this work, a simple DFT algorithm is chosen as the estimator in the PMU implemented in real-time. The selected method consists of applying the algorithm to a single-phase signal. Given that the objective of this research involves the characterization of a virtual PMU, then the use of a strictly three-phase algorithm requiring more resources is discarded—for instance, Clarke transformation-based [79] and positive sequence estimation algorithms [80]. Moreover, this choice means that the algorithm could be easily duplicated for a real-case three-phase signal. In addition, as above-mentioned, this choice implies a limited computational burden aiming at having the least impact on the cores of the CPU of the RTS, which would be able to perform the phasor evaluation within fixed time steps.

Even though parallelization could be possible, our choice of a single-phase DFT algorithm is also based on the objective of this paper. Indeed, the proposed work aims at highlighting the importance of the characterization of virtual PMUs for HIL applications; hence, the achievement of the best estimating algorithm is out of the scope of this research.

Finally, according to [47], PMUs can be classified into two classes of performances: P-class (protection applications requiring a fast response) and M-class (measurement applications requiring high accuracy). The latter is considered the type of virtual device implemented in the RTS, considering that it also includes the performance limits of the P-class.

Tests that will be described in the next Section 4.1 are based on [47], which specifies both steady-state and the dynamic performance compliance criteria for each class of PMUs.

## 4. Tests and Results

This section contains the core of the work. As previously mentioned, a virtual PMU is tested to highlight its significant contribution to the overall uncertainty of a system. The set of tests designed for assessing the performance of a virtual PMU considering the RTS contribution is fully described in accordance with the standard IEC 60255 [47]. The goal of the tests is an increased awareness by more RTSs users in performing preliminary characterization of their virtual models.

### 4.1. PMU Testing

**Figure 8:**
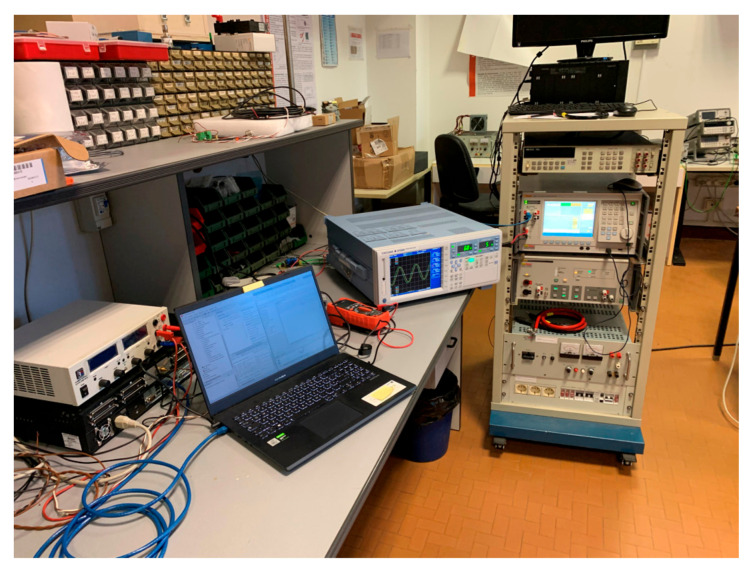
Picture taken of the laboratory environment during the RTS testing.

The tests performed on the virtual PMU hosted inside the RTS aim to assess the amplitude, the phase, the frequency, and the distorted signals. For each aspect, a specific test has been performed. For all tests, the time step of the simulator is 50 μs, and the sampling frequency of the analog input DAQ is 20 kSa/s. A picture of the test setup is shown in Figure 8. To better clarify the testing idea, the virtual PMU inside the RTS is tested according to the PMU standard [47]. This is to treat the virtual PMU like a physical one when its testing is concerned. Therefore, amplitude, phase, harmonic, and frequency tests are described in what follows.

#### 4.1.1. Amplitude Tests

The amplitude tests have been designed considering both the current and voltage limits defined in [47]. In fact, the current variation is wider (from 10% to 200%); therefore, the RTS DAQ max input (10 V) has been associated with 200% of the rated signal. Consequently, five tests, referred to as A1 to A5, from 10% to 200% of the rated signal, are designed and described in Table 11. The table contains the phase, the frequency, and the amplitude (peak and RMS) of the adopted signals.

#### 4.1.2. Frequency Tests

In accordance with [47], nine different tests, referred to as F1 to F9, have been designed for testing the virtual PMU behavior vs. frequency. They are collected in Table 12. Adopting 100% of the rated signal, nine frequency values from 48 Hz to 52 Hz, with steps of 0.5 Hz, have been used. For the frequency tests, the initial phase of the signals is always set to zero.

#### 4.1.3. Harmonic Tests

The aim of these tests is to verify the performance of a PMU when a single harmonic component is superimposed to the main frequency signal. In detail, the harmonic component has an amplitude corresponding to the 10% of the main signal.

The defined tests are listed in Table 13. For each test, referred to as H1 to H16, the table contains the amplitude of both the main signal and the harmonic component. The tested harmonic components range from the third to the forty-ninth odd harmonics.

#### 4.1.4. Phase Tests

Typical laboratory tests use 0 as the initial phase. However, considering real applications, a set of tests tackling the performance of the virtual PMUs when the phase is not null is necessary. Therefore, Table 14 presents 11 tests, referred to as P1 to P11, in which the phase varies from 0° to 100° with steps of 10°. The table also contains the amplitude of the signal (always 100% of the rated) and the frequency (50 Hz).

### 4.2. Tests Results

Each test described in Section 4.1 had a duration of 20 s, during which the quantities have been collected and then averaged to obtain the final results. The average values and their standard deviation of the mean are collected in Table 15, Table 16, Table 17 and Table 18 for the amplitude, frequency, harmonic, and phase, respectively. Every table contains the RMS value of the measured voltage RMS, its standard deviation of the mean $\sigma_{RMS}$, the phase, its standard deviation of the mean $\sigma_{Ph}$, the measured frequency, its standard deviation of the mean $\sigma_{Fr}$, the ROCOF, and its standard deviation of the mean $\sigma_{R}$.

From the tables, it can be observed that the results are quite coherent, and, in particular, the standard deviation of the mean (the absolute one) is always in the order of 10^−8^, 10^−7^, 10^−7^, and 10^−5^ for the amplitude, phase, frequency, and ROCOF, respectively.

**Figure 9:**
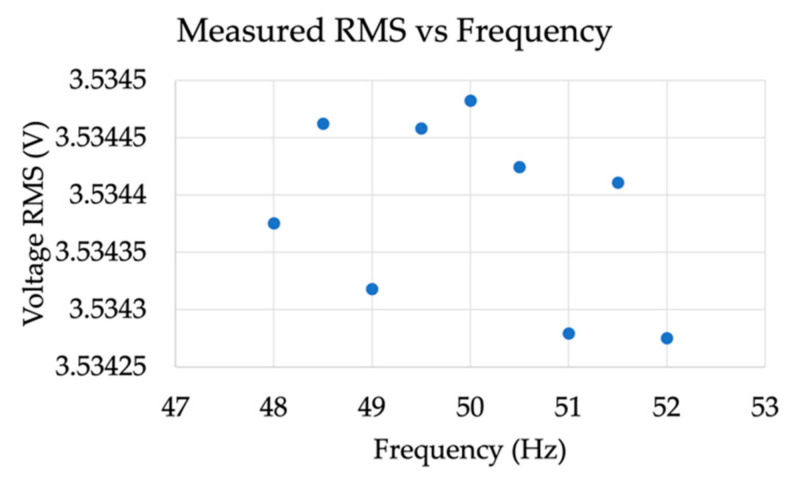
RMS vs. frequency of results in Table 16.

**Figure 10:**
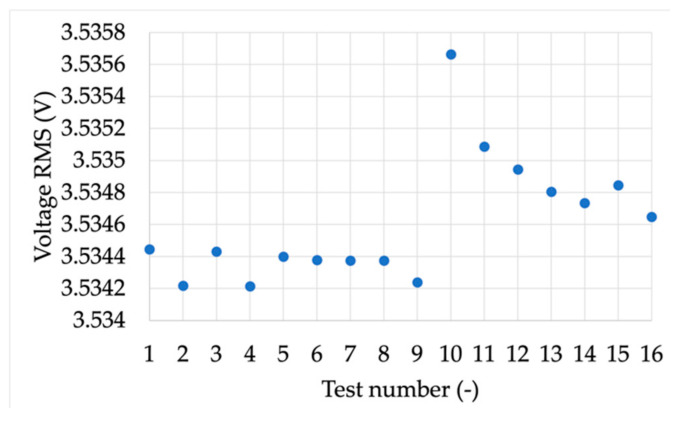
RMS for each harmonic test (Table 17).

For the sake of readability of the results, Figure 9 and Figure 10 show the RMS vs. frequency and the RMS for each harmonic test taken from Table 16 and Table 17, respectively.

Despite the good preliminary results, it is necessary to evaluate them according to the indices defined in [47]. Furthermore, the propagation of the uncertainty process is fundamental to quantify and assess the accuracy of the presented results.

To this purpose, the results presented in Table 15, Table 16, Table 17 and Table 18, along with the reference values provided and set by the reference calibrator, are used to compute the TVE, FE, and RFE for each of the performed tests.

The obtained indices are collected in Table 19, Table 20, Table 21 and Table 22 for the amplitude, frequency, harmonics, and phase, respectively. The tables are coherent among each other, and they contain: TVE, FE, RFE, and their combined uncertainties $u_{TVE}$, $u_{FE}$, and $u_{RFE}$, respectively.

Note that the combined uncertainties have been computed considering (i) the standard deviation of the measured quantities (Table 15, Table 16, Table 17 and Table 18), (ii) the standard deviation of the computed indices, and (iii) the formula used to compute the index. Concerning the last aspect, the uncertainty propagation is straightforward in the case of FE and RFE. In fact, the mathematical operation is subtraction. On the contrary, the TVE expression is quite complicated compared to the previous two; therefore, the Monte Carlo method has been used to obtain the uncertainty associated with TVE (100,000 trials).

Starting from Table 19, different comments arise. In the table, but also for the flowing ones, when the mean value is lower than the obtained combined uncertainty, the choice has been of leaving the full number and not putting zero. Such a choice is supported by the aim of showing the discrepancies between the order of magnitude of the quantity and its combined uncertainty. Therefore, the significant digits are coherent only in the case of combined uncertainty lower than the mean value.

Another comment involves the indices. While FE and RFE are always below the limits defined by [47] (even if a test involving the amplitude variation is not defined), the TVE exceeds the limits in test A5. Such a test is the one with the smallest input test signal (0.1 V). It is important then to understand the capabilities of the RTS system before using them for simulating purposes. As in the case of the tests, it is demonstrated from the results of Table 19 that the characterization of the virtual PMU in terms of amplitude is fundamental to know and correct the values during normal operations.

For the results in Table 20, what is stated for RFE and FE in the case of Table 19 still applies. However, the TVE does not always remain within the limits of the standard. In particular, it exceeds 1% in test F2 and is 0.921 in test F3. On average, all frequency test results are not really satisfactory, and the reason can be attributed to the acquisition process of the RTS.

**Figure 11:**
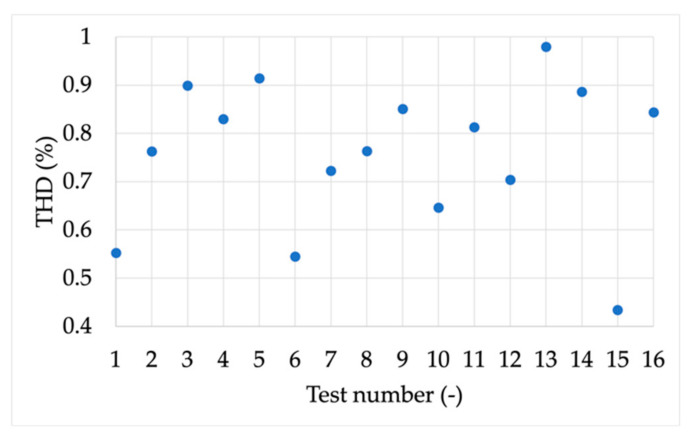
THD obtained in each harmonic test from H1 to H16.

Table 21 lists the indices computed in the case of the harmonic tests. From the results, it emerges that FE and RFE are far below the defined limits. As for TVE, it is below the 1% limits in all tests, but on average is always higher than 0.6%. For a better overview of the TVE results, considering the number of digits involved, Figure 11 has been used.

Identical comments can be made for the results in Table 22, which contain the indices computed for the phase tests.

**Figure 12:**
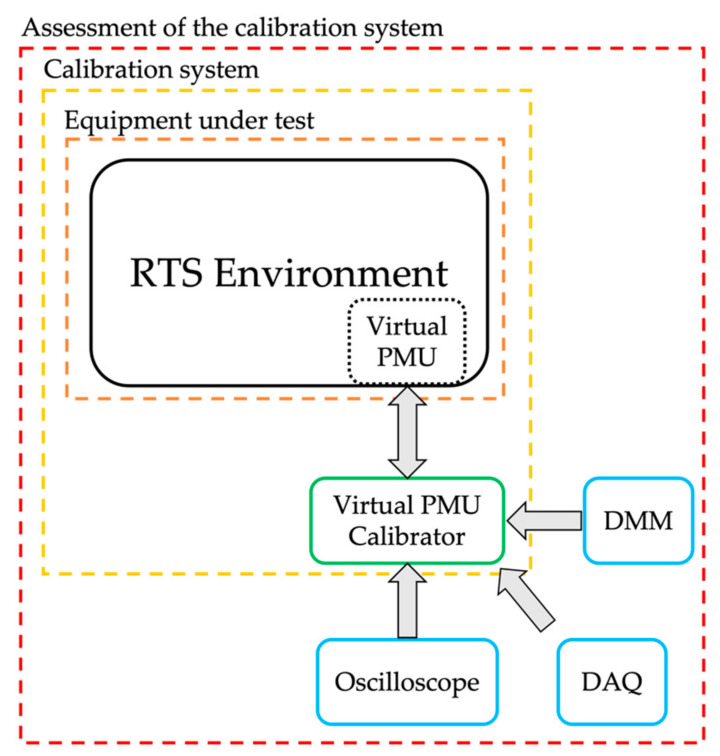
Block diagram of the complete research.

In light of all results presented in the previous tables, it can be concluded that the characterization of a virtual PMU inside an RTS environment is fundamental for accuracy purposes (see Figure 12 for a simplified block diagram of the research). In fact, what was obtained clearly emphasizes the need for a priori knowledge of the performance of each component to be used within a measurement setup. This is to avoid unnecessary propagation of uncertainties from one component to another.

In the specific case considered, the single contribution of the RTS is not negligible, and in some cases, the limits defined by the standard [47] are not satisfied. In addition, the results must be assessed considering that the source of the test signals was generated by an accurate calibrator (see Section 2.3.6). Consequently, if real sensors with typical accuracy ranging from accuracy class 0.5–1 are considered, the overall uncertainty propagated in the final results would significantly increase.

## 5. Conclusions

The use of real-time simulators among researchers and utilities is increasing day after day. This allows enhancing the simulation capabilities, including the possibility to recreate complete digital models of the power network. However, the RTSs must ensure a high level of accuracy for their results to be reliable enough for the final users. This aspect is not always considered and sufficiently treated. The article has the aim of emphasizing and supporting, with rigorous experimental activity, the lack of methodology found in the literature. A virtual PMU is then characterized by testing it like a physical PMU, hence by using the same tests defined in the dedicated standard. Furthermore, to complete the discussion, the complete characterization process of a PMU calibrator is described. The main result of the virtual PMU characterization is not the index remaining within or exceeding the limits. The main result is the experimental proof that the preliminary characterization of the virtual PMU is mandatory when an RTS environment must be used for simulating power networks. Such a result is confirmed by all the performed tests, and its importance can be extended to all activities involving an RTS, not necessarily correlated to power networks or electrical engineering in general.

## Figures and Tables

**Table 1:** Calibrator’s analog output DAC specifications.

DAC Properties	Value
Resolution	16 bit
Full range	±10 V
Max sampling rate	900 kSa/s

**Table 2:** GPS disciplined oscillator specifications.

GPS Properties	Value
PPS accuracy	15 ns (one sigma)
10 MHz clock accuracy	$1.16\times 10^{-12}$ Hz (one day average)
10 MHz stability	See Allan deviation graph in [63]

**Table 3:** Steady-state signal magnitude test points.

Set Point (% of Nominal Value)	Peak Set Point (V)	RMS Set Point V *X*_1_
10	0.5	≈0.35
20	1	≈0.71
50	2.5	≈1.77
100	5	≈3.54
120	6	≈4.24
150	7.5	≈5.30
200	10	≈7.07

**Table 4:** Steady-state harmonic disturbance test points.

	50 Hz Component (% of Nominal Value)	Harmonic Component Order *h*	Harmonic Component Magnitude (% of 50 Hz Signal Nominal Value)
Single harmonic component test signal $X_{h}$	0	From 2 to 50	10
Standard harmonic distortion test signal $X_{1+h}$	100	2	10
		3	10
		5	10
		7	10
		11	10
		20	10
		30	10
		50	10

**Table 5:** Steady-state signal magnitude characterization results.

$X_{1}{\;\mathrm{RMS}}^{*}.$ (V)	$\mu_{X_{1}\;\mathrm{RMS}}$ (V)	$\sigma_{X_{1}\;\mathrm{RMS}}$ (V)	$u_{{A,\;X}_{1}\;\mathrm{RMS}}$ (V)	$u_{{B,\;X}_{1}\;\mathrm{RMS}}$ (V)	$\Delta_{X_{1}\;\mathrm{RMS}}$ (V)
0.3535534	0.3535314	$2\times 10^{-6}$	$3\times 10^{-7}$	$3\times 10^{-5}$	$2\times 10^{-5}$
0.7071068	0.7070302	$2\times 10^{-6}$	$3\times 10^{-7}$	$4\times 10^{-5}$	$8\times 10^{-5}$
1.767767	1.767642	$3\times 10^{-5}$	$4\times 10^{-6}$	$2\times 10^{-4}$	$1\times 10^{-4}$
3.535534	3.535152	$2\times 10^{-5}$	$3\times 10^{-6}$	$3\times 10^{-4}$	$4\times 10^{-4}$
4.242641	4.242152	$2\times 10^{-5}$	$3\times 10^{-6}$	$3\times 10^{-4}$	$5\times 10^{-4}$
5.303301	5.302879	$2\times 10^{-5}$	$3\times 10^{-6}$	$3\times 10^{-4}$	$4\times 10^{-4}$
7.071068	7.070681	$2\times 10^{-5}$	$3\times 10^{-6}$	$4\times 10^{-4}$	$4\times 10^{-4}$

**Table 6:** Steady-state single harmonic component signal characterization results.

h.	$X_{h}{\;\mathrm{RMS}}^{*}$ (V)	$\mu_{X_{h}\;\mathrm{RMS}}$ (V)	$\sigma_{X_{h}\;\mathrm{RMS}}$ (V)	$u_{{A,\;X}_{h}\;\mathrm{RMS}}$ (V)	$u_{{B,\;X}_{h}\;\mathrm{RMS}}$ (V)	$\Delta_{X_{h}\;\mathrm{RMS}}$ (V)
5	0.3535534	0.3535502	$1\times 10^{-6}$	$2\times 10^{-7}$	$3\times 10^{-5}$	$3\times 10^{-6}$
50		0.3535131	$1\times 10^{-6}$	$1\times 10^{-7}$	$4\times 10^{-5}$	$4\times 10^{-5}$

**Table 7:** Steady-state standard harmonic disturbance signal characterization results.

h.	$X_{1+h}{\;\mathrm{RMS}}^{*}$ (V)	$\mu_{X_{1+h}\;\mathrm{RMS}}$ (V)	$\sigma_{X_{1+h}\;\mathrm{RMS}}$ (V)	$u_{{A,\;X}_{1+h}\;\mathrm{RMS}}$ (V)	$u_{{B,\;X}_{1+h}\;\mathrm{RMS}}$ (V)	$\Delta_{X_{1+h}\;\mathrm{RMS}}$ (V)
2	3.5531676	3.552858	$2\times 10^{-5}$	$3\times 10^{-6}$	$3\times 10^{-4}$	$2.8\times 10^{-4}$
3		3.552888	$2\times 10^{-5}$	$3\times 10^{-6}$	$3\times 10^{-4}$	$2.8\times 10^{-4}$
5		3.552913	$2\times 10^{-5}$	$2\times 10^{-6}$	$3\times 10^{-4}$	$2.5\times 10^{-4}$
7		3.552886	$2\times 10^{-5}$	$3\times 10^{-6}$	$3\times 10^{-4}$	$2.8\times 10^{-4}$
11		3.552876	$2\times 10^{-5}$	$3\times 10^{-6}$	$4\times 10^{-4}$	$2.9\times 10^{-4}$
20		3.552886	$2\times 10^{-5}$	$3\times 10^{-6}$	$4\times 10^{-4}$	$2.8\times 10^{-4}$
30		3.552893	$2\times 10^{-5}$	$3\times 10^{-6}$	$4\times 10^{-4}$	$2.7\times 10^{-4}$
50		3.552720	$7\times 10^{-5}$	$9\times 10^{-6}$	$4\times 10^{-4}$	$4.5\times 10^{-4}$

**Table 8:** Steady-state signal frequency characterization results.

$f^{*}.$ (Hz)	$\mu_{f}$ (Hz)	$u_{A,\;f}$ (Hz)	$u_{B,\;f}$ (Hz)	$\Delta_{f}$ (Hz)	$\mu_{\mathrm{ROCOF}}$ (Hz)	$u_{A,\;\mathrm{ROCOF}}$ (Hz)	$u_{B,\;\mathrm{ROCOF}}$ (Hz)	$\delta_{\max-\min}$ (Hz)
45.0	45.000000	$1\times 10^{-6}$	$3\times 10^{-5}$	$1.5\times 10^{-7}$	0	$2\times 10^{-6}$	$4\times 10^{-5}$	$8\times 10^{-5}$
45.5	45.500002	$1\times 10^{-6}$	$3\times 10^{-5}$	$1.5\times 10^{-6}$	− $3\times 10^{-7}$	$1\times 10^{-6}$	$4\times 10^{-5}$	$5\times 10^{-5}$
46.0	46.000001	$1\times 10^{-6}$	$3\times 10^{-5}$	$1.1\times 10^{-6}$	$2\times 10^{-8}$	$1\times 10^{-6}$	$4\times 10^{-5}$	$6\times 10^{-5}$
46.5	46.500001	$1\times 10^{-6}$	$3\times 10^{-5}$	$1.4\times 10^{-6}$	$9\times 10^{-8}$	$1\times 10^{-6}$	$4\times 10^{-5}$	$6\times 10^{-5}$
47.0	47.000001	$1\times 10^{-6}$	$3\times 10^{-5}$	$1.1\times 10^{-6}$	$2\times 10^{-7}$	$1\times 10^{-6}$	$4\times 10^{-5}$	$5\times 10^{-5}$
47.5	47.500002	$1\times 10^{-6}$	$3\times 10^{-5}$	$1.5\times 10^{-6}$	$4\times 10^{-7}$	$2\times 10^{-6}$	$4\times 10^{-5}$	$6\times 10^{-5}$
48.0	48.000001	$1\times 10^{-6}$	$3\times 10^{-5}$	$1.4\times 10^{-6}$	$2\times 10^{-7}$	$1\times 10^{-6}$	$4\times 10^{-5}$	$5\times 10^{-5}$
48.5	48.500006	$1\times 10^{-6}$	$3\times 10^{-5}$	$5.6\times 10^{-6}$	− $1\times 10^{-7}$	$1\times 10^{-6}$	$4\times 10^{-5}$	$6\times 10^{-5}$
49.0	49.000003	$1\times 10^{-6}$	$3\times 10^{-5}$	$2.9\times 10^{-6}$	− $2\times 10^{-7}$	$1\times 10^{-6}$	$4\times 10^{-5}$	$5\times 10^{-5}$
49.5	49.500000	$1\times 10^{-6}$	$3\times 10^{-5}$	$4.6\times 10^{-7}$	− $4\times 10^{-8}$	$2\times 10^{-6}$	$4\times 10^{-5}$	$7\times 10^{-5}$
50.0	50.000002	$8\times 10^{-7}$	$3\times 10^{-5}$	$2.3\times 10^{-6}$	0	$1\times 10^{-6}$	$4\times 10^{-5}$	$4\times 10^{-5}$
50.5	50.500000	$1\times 10^{-6}$	$3\times 10^{-5}$	− $4.1\times 10^{-8}$	− $5\times 10^{-8}$	$1\times 10^{-6}$	$4\times 10^{-5}$	$6\times 10^{-5}$
51.0	51.000001	$1\times 10^{-6}$	$3\times 10^{-5}$	$1.3\times 10^{-6}$	$1\times 10^{-7}$	$2\times 10^{-6}$	$4\times 10^{-5}$	$8\times 10^{-5}$
51.5	51.500007	$1\times 10^{-6}$	$3\times 10^{-5}$	$6.6\times 10^{-6}$	$2\times 10^{-7}$	$2\times 10^{-6}$	$4\times 10^{-5}$	$6\;\times 10^{-5}$
52.0	52.000003	$1\times 10^{-6}$	$3\times 10^{-5}$	$3.1\times 10^{-6}$	− $2\times 10^{-8}$	$2\times 10^{-6}$	$4\times 10^{-5}$	$7\times 10^{-5}$
52.5	52.500004	$1\times 10^{-6}$	$3\times 10^{-5}$	$4.1\times 10^{-6}$	− $1\times 10^{-7}$	$2\times 10^{-6}$	$4\times 10^{-5}$	$7\times 10^{-5}$
53.0	53.000006	$1\times 10^{-6}$	$3\times 10^{-5}$	$5.6\times 10^{-6}$	$8\times 10^{-8}$	$1\times 10^{-6}$	$4\times 10^{-5}$	$6\times 10^{-5}$
53.5	53.500004	$1\times 10^{-6}$	$3\times 10^{-5}$	$4.1\times 10^{-6}$	− $8\times 10^{-8}$	$1\times 10^{-6}$	$4\times 10^{-5}$	$6\times 10^{-5}$
54.0	54.000005	$1\times 10^{-6}$	$3\times 10^{-5}$	$5.3\times 10^{-6}$	$5\times 10^{-8}$	$1\times 10^{-6}$	$4\times 10^{-5}$	$6\times 10^{-5}$
54.5	54.500001	$1\times 10^{-6}$	$3\times 10^{-5}$	$1.3\times 10^{-6}$	− $2\times 10^{-8}$	$1\times 10^{-6}$	$4\times 10^{-5}$	$5\times 10^{-5}$
55.0	55.000004	$1\times 10^{-6}$	$3\times 10^{-5}$	$4.0\times 10^{-6}$	$7\times 10^{-8}$	$2\times 10^{-6}$	$4\times 10^{-5}$	$6\times 10^{-5}$

**Table 9:** “Actual zero phase displacement” characterization results.

$\mu_{\phi 0}\;(\mathrm{rad})$	$u_{A,\;\phi 0}\;(\mathrm{rad})$
1.27 $\times \;10^{-7}$	4 $\times \;10^{-9}$

**Table 10:** Phase displacement characterization results.

$\phi_{\mathrm{OUT}}^{*}\;(\mathrm{rad})$	$\phi_{\mathrm{OUT}2}^{*}\;(\mathrm{rad})$	$\mu_{\phi }\;(\mathrm{rad})$	$u_{A,\;\phi }\;(\mathrm{rad})$	$\Delta_{\phi }\;(\mathrm{rad})$
0	0	$2.690\times 10^{-6}$	$4\times 10^{-9}$	− $2.7\times 10^{-6}$
0.523598776	0	0.5236015	$2\times 10^{-7}$	− $2.8\times 10^{-6}$
0.785398163	0	0.7854010	$3\times 10^{-7}$	− $2.9\times 10^{-6}$
1.570796327	0	1.5707995	$4\times 10^{-7}$	− $3.2\times 10^{-6}$

**Table 11:** Settings of the amplitude tests.

Test Name	Peak (V)	RMS (V)	% of Rated (%)	Phase (rad)	Frequency (Hz)
A1	10	7.0710	200	0	50
A2	5	3.5355	100	0	50
A3	2.5	1.7677	50	0	50
A4	1	0.7071	20	0	50
A5	0.1	0.0707	10	0	50

**Table 12:** Settings of the frequency tests.

Test Name	Peak (V)	RMS (V)	% of Rated (%)	Phase (rad)	Frequency (Hz)
F1	5	3.5355	100	0	48
F2	5	3.5355	100	0	48.5
F3	5	3.5355	100	0	49
F4	5	3.5355	100	0	49.5
F5	5	3.5355	100	0	50
F6	5	3.5355	100	0	50.5
F7	5	3.5355	100	0	51
F8	5	3.5355	100	0	51.5
F9	5	3.5355	100	0	52

**Table 13:** Settings of the harmonic tests.

Test Name	Peak (V)	RMS (V)	% of Rated (%)	Order (-)	% of 50 Hz Comp (%)
H1	5	3.5355	100	3	10
H2	5	3.5355	100	5	10
H3	5	3.5355	100	7	10
H4	5	3.5355	100	9	10
H5	5	3.5355	100	11	10
H6	5	3.5355	100	15	10
H7	5	3.5355	100	19	10
H8	5	3.5355	100	21	10
H9	5	3.5355	100	25	10
H10	5	3.5355	100	29	10
H11	5	3.5355	100	31	10
H12	5	3.5355	100	35	10
H13	5	3.5355	100	39	10
H14	5	3.5355	100	41	10
H15	5	3.5355	100	45	10
H16	5	3.5355	100	49	10

**Table 14:** Settings of the phase tests.

Test Name	Peak (V)	RMS (V)	% of Rated (%)	Frequency (Hz)	Phase (°)	Phase (rad)
P1	5	3.5355	100	50	0	0
P2	5	3.5355	100	50	10	0.1745
P3	5	3.5355	100	50	20	0.3490
P4	5	3.5355	100	50	30	0.5235
P5	5	3.5355	100	50	40	0.6981
P6	5	3.5355	100	50	50	0.8726
P7	5	3.5355	100	50	60	1.0471
P8	5	3.5355	100	50	70	1.2217
P9	5	3.5355	100	50	80	1.3962
P10	5	3.5355	100	50	90	1.5707
P11	5	3.5355	100	50	100	1.7453

**Table 15:** Measurement results of the amplitude tests.

**Test Name**	RMS (V)	$\sigma_{RMS}\;(V)$	Phase (rad)	$\sigma_{Ph}\;(\mathrm{rad})$	Frequency (Hz)	$\sigma_{Fr}\;(\mathrm{Hz})$	ROCOF (Hz/s)	$\sigma_{R}\;(\mathrm{Hz}/s)$
A1	7.0692976	$1\times 10^{-7}$	0.0054920	$4\times 10^{-7}$	50.0000000	$3\times 10^{-7}$	$3.24\times 10^{-7}$	$2\times 10^{-5}$
A2	3.53433398	$9\times 10^{-8}$	0.0088586	$4\times 10^{-7}$	50.0000002	$3\times 10^{-7}$	$1.77\times 10^{-6}$	$3\times 10^{-5}$
A3	1.76701027	$8\times 10^{-8}$	0.0093632	$4\times 10^{-7}$	50.0000000	$3\times 10^{-7}$	$-4.19\times 10^{-6}$	$7\times 10^{-5}$
A4	0.70625170	$8\times 10^{-8}$	−0.0035345	$3\times 10^{-7}$	50.0000004	$3\times 10^{-7}$	$1.25\times 10^{-6}$	$2\times 10^{-4}$
A5	0.06985583	$8\times 10^{-8}$	0.007932	$3\times 10^{-6}$	49.999975	$2\times 10^{-6}$	$-1.35\times 10^{-5}$	$2\times 10^{-3}$

**Table 16:** Measurement results of the frequency tests.

**Test Name**	**RMS (V)**	$\sigma_{RMS}$ **(V)**	**Phase (rad)**	$\sigma_{Ph}$ **(rad)**	**Frequency (Hz)**	$\sigma_{Fr}$ **(Hz)**	**ROCOF (Hz/s)**	$\sigma_{R}$ **(Hz/s)**
F1	3.5343752	$1\times 10^{-7}$	0.0073137	$4\times 10^{-7}$	48.0000000	$3\times 10^{-7}$	$-9.19\times 10^{-7}$	$5\times 10^{-5}$
F2	3.5344621	$1\times 10^{-7}$	0.0119328	$4\times 10^{-7}$	48.4999925	$2\times 10^{-7}$	$3.94\times 10^{-5}$	$5\times 10^{-5}$
F3	3.53431802	$9\times 10^{-8}$	0.0092100	$4\times 10^{-7}$	48.9999999	$3\times 10^{-7}$	$-1.17\times 10^{-6}$	$5\times 10^{-5}$
F4	3.5344581	$1\times 10^{-7}$	0.008737	$1\times 10^{-6}$	49.5000027	$3\times 10^{-7}$	$-2.72\times 10^{-6}$	$5\times 10^{-5}$
F5	3.53448223	$8\times 10^{-8}$	−0.0031022	$4\times 10^{-7}$	49.9999971	$3\times 10^{-7}$	$4.90\times 10^{-5}$	$3\times 10^{-5}$
F6	3.5344243	$1\times 10^{-7}$	−0.0027930	$6\times 10^{-7}$	50.4999894	$2\times 10^{-7}$	$-3.13\times 10^{-7}$	$5\times 10^{-5}$
F7	3.5342794	$1\times 10^{-7}$	0.0034803	$5\times 10^{-7}$	50.9999988	$3\times 10^{-7}$	$1.24\times 10^{-5}$	$5\times 10^{-5}$
F8	3.5344108	$1\times 10^{-7}$	0.0072208	$4\times 10^{-7}$	51.4999955	$3\times 10^{-7}$	$9.18\times 10^{-7}$	$6\times 10^{-5}$
F9	3.5342751	$1\times 10^{-7}$	−0.0047451	$4\times 10^{-7}$	52.0000001	$3\times 10^{-7}$	$-2.46\times 10^{-6}$	$6\times 10^{-5}$

**Table 17:** Measurement results of the harmonic tests.

**Test Name**	RMS (V)	$\sigma_{RMS}\;(V)$	Phase (rad)	$\sigma_{Ph}\;(\mathrm{rad})$	Frequency (Hz)	$\sigma_{Fr}\;(\mathrm{Hz})$	ROCOF (Hz/s)	$\sigma_{R}\;(\mathrm{Hz}/s)$
H1	3.53444568	$8\times 10^{-8}$	0.0055126	$4\times 10^{-7}$	50.0000008	$3\times 10^{-7}$	$-1.60\times 10^{-6}$	$3\times 10^{-5}$
H2	3.53421753	$9\times 10^{-8}$	0.0076177	$8\times 10^{-7}$	50.0000070	$3\times 10^{-7}$	$-1.51\times 10^{-6}$	$4\times 10^{-5}$
H3	3.53443023	$9\times 10^{-8}$	0.0089869	$7\times 10^{-7}$	50.0000075	$3\times 10^{-7}$	$-1.17\times 10^{-6}$	$4\times 10^{-5}$
H4	3.5342145	$1\times 10^{-7}$	0.0082917	$9\times 10^{-7}$	50.0000136	$3\times 10^{-7}$	$1.65\times 10^{-6}$	$4\times 10^{-5}$
H5	3.53439991	$8\times 10^{-8}$	0.0091382	$5\times 10^{-7}$	49.9999942	$2\times 10^{-7}$	$1.99\times 10^{-6}$	$4\times 10^{-5}$
H6	3.5343785	$1\times 10^{-7}$	0.0054355	$5\times 10^{-7}$	50.0000072	$3\times 10^{-7}$	$2.26\times 10^{-6}$	$4\times 10^{-5}$
H7	3.5343746	$1\times 10^{-7}$	0.0072207	$4\times 10^{-7}$	50.0000003	$3\times 10^{-7}$	$3.37\times 10^{-8}$	$4\times 10^{-5}$
H8	3.5343741	$2\times 10^{-7}$	0.0076282	$4\times 10^{-7}$	50.0000000	$3\times 10^{-7}$	$3.14\times 10^{-7}$	$4\times 10^{-5}$
H9	3.5342377	$2\times 10^{-7}$	0.0085004	$9\times 10^{-7}$	49.9999873	$2\times 10^{-7}$	$-2.13\times 10^{-7}$	$5\times 10^{-5}$
H10	3.5356639	$1\times 10^{-7}$	0.005984	$6\times 10^{-6}$	50.0000197	$7\times 10^{-7}$	$-4.50\times 10^{-6}$	$7\times 10^{-5}$
H11	3.5350865	$1\times 10^{-7}$	0.0081300	$5\times 10^{-7}$	50.0000089	$3\times 10^{-7}$	$2.50\times 10^{-6}$	$5\times 10^{-5}$
H12	3.5349436	$1\times 10^{-7}$	0.0070349	$3\times 10^{-7}$	49.9999980	$2\times 10^{-7}$	$-2.99\times 10^{-6}$	$5\times 10^{-5}$
H13	3.5348042	$2\times 10^{-7}$	0.0097929	$3\times 10^{-7}$	49.9999977	$2\times 10^{-7}$	$-1.06\times 10^{-6}$	$5\times 10^{-5}$
H14	3.5347333	$1\times 10^{-7}$	0.008859	$2\times 10^{-6}$	50.0000258	$3\times 10^{-7}$	$7.53\times 10^{-7}$	$6\times 10^{-5}$
H15	3.53484464	$9\times 10^{-8}$	0.0043363	$4\times 10^{-7}$	49.9999995	$3\times 10^{-7}$	$9.23\times 10^{-7}$	$5\times 10^{-5}$
H16	3.5346480	$2\times 10^{-7}$	0.008433	$1\times 10^{-6}$	50.0000127	$3\times 10^{-7}$	$-1.27\times 10^{-6}$	$6\times 10^{-5}$

**Table 18:** Measurement results of the phase tests.

**Test Name**	RMS (V)	$\sigma_{RMS}\;(V)$	Phase (rad)	$\sigma_{Ph}\;(\mathrm{rad})$	Frequency (Hz)	$\sigma_{Fr}\;(\mathrm{Hz})$	ROCOF (Hz/s)	$\sigma_{R}\;(\mathrm{Hz}/s)$
P1	3.53407799	$9\times 10^{-8}$	0.0113214	$8\times 10^{-7}$	50.0000045	$3\times 10^{-7}$	$2.46\times 10^{-7}$	$3\times 10^{-5}$
P2	3.53414587	$8\times 10^{-8}$	0.1757891	$4\times 10^{-7}$	49.9999982	$3\times 10^{-7}$	$1.93\times 10^{-6}$	$3\times 10^{-5}$
P3	3.53423390	$8\times 10^{-8}$	0.3574863	$4\times 10^{-7}$	49.9999988	$3\times 10^{-7}$	$-4.54\times 10^{-6}$	$3\times 10^{-5}$
P4	3.53424212	$8\times 10^{-8}$	0.5307076	$4\times 10^{-7}$	49.9999991	$3\times 10^{-7}$	$-1.11\times 10^{-6}$	$3\times 10^{-5}$
P5	3.5341987	$1\times 10^{-7}$	0.7064876	$7\times 10^{-7}$	50.0000064	$3\times 10^{-7}$	$-1.29\times 10^{-5}$	$3\times 10^{-5}$
P6	3.53416886	$9\times 10^{-8}$	0.8733776	$4\times 10^{-7}$	49.9999999	$3\times 10^{-7}$	$-9.72\times 10^{-6}$	$3\times 10^{-5}$
P7	3.53417573	$9\times 10^{-8}$	1.0548074	$4\times 10^{-7}$	49.9999991	$3\times 10^{-7}$	$-3.91\times 10^{-6}$	$3\times 10^{-5}$
P8	3.5340813	$1\times 10^{-7}$	1.224595	$2\times 10^{-6}$	50.0000105	$4\times 10^{-7}$	$-5.17\times 10^{-6}$	$4\times 10^{-5}$
P9	3.5340916	$1\times 10^{-7}$	1.4034948	$4\times 10^{-7}$	49.9999980	$3\times 10^{-7}$	$-7.36\times 10^{-6}$	$3\times 10^{-5}$
P10	3.5340525	$1\times 10^{-7}$	1.5760936	$8\times 10^{-7}$	49.9999852	$3\times 10^{-7}$	$-5.18\times 10^{-6}$	$3\times 10^{-5}$
P11	3.53407339	$8\times 10^{-8}$	1.7542494	$4\times 10^{-7}$	49.9999982	$3\times 10^{-7}$	$-9.10\times 10^{-6}$	$3\times 10^{-5}$

**Table 19:** Indices of the amplitude tests.

**Test Name**	TVE (%)	$u_{TVE}\;(-)$	FE (Hz)	$\sigma_{FE}\;(\mathrm{Hz})$	RFE (Hz/s)	$\sigma_{RFE}\;(\mathrm{Hz}/s)$
A1	0.54970	$3\times 10^{-7}$	$-2.89\times 10^{-8}$	$1\times 10^{-6}$	$3.42\times 10^{-7}$	$2\times 10^{-5}$
A2	0.31161	$3\times 10^{-7}$	$-3\times 10^{-6}$	$1\times 10^{-6}$	$5\times 10^{-5}$	$4\times 10^{-5}$
A3	0.93710	$3\times 10^{-7}$	$-3.41\times 10^{-8}$	$1\times 10^{-6}$	$-4.19\times 10^{-6}$	$7\times 10^{-5}$
A4	0.37348	$2\times 10^{-7}$	$3.69\times 10^{-7}$	$1\times 10^{-6}$	$1.25\times 10^{-6}$	$2\times 10^{-4}$
A5	1.4512	$9\times 10^{-7}$	$-2.5\times 10^{-5}$	$2\times 10^{-6}$	$-1.35\times 10^{-5}$	$3\times 10^{-4}$

**Table 20:** Measurement results of the frequency tests.

**Test Name**	TVE (%)	$u_{TVE}\;(-)$	FE (Hz)	$\sigma_{FE}\;(\mathrm{Hz})$	RFE (Hz/s)	$\sigma_{RFE}\;(\mathrm{Hz}/s)$
F1	0.73198	$3\times 10^{-7}$	$-4.45\times 10^{-8}$	$1\times 10^{-6}$	$-9.19\times 10^{-7}$	$5\times 10^{-5}$
F2	1.19348	$3\times 10^{-7}$	$-7\times 10^{-6}$	$1\times 10^{-6}$	$4\times 10^{-5}$	$5\times 10^{-5}$
F3	0.92148	$3\times 10^{-7}$	$-5.35\times 10^{-8}$	$1\times 10^{-6}$	$-1.17\times 10^{-6}$	$5\times 10^{-5}$
F4	0.87410	$6\times 10^{-7}$	$3\times 10^{-6}$	$2\times 10^{-6}$	$-2.72\times 10^{-6}$	$5\times 10^{-5}$
F5	0.31161	$3\times 10^{-7}$	$-3\times 10^{-6}$	$1\times 10^{-6}$	$5\times 10^{-5}$	$4\times 10^{-5}$
F6	0.28105	$4\times 10^{-7}$	$-1.1\times 10^{-5}$	$1\times 10^{-6}$	$-3.13\times 10^{-7}$	$5\times 10^{-5}$
F7	0.34979	$4\times 10^{-7}$	$-1\times 10^{-6}$	$2\times 10^{-6}$	$1\times 10^{-5}$	$5\times 10^{-5}$
F8	0.72267	$3\times 10^{-7}$	$-4\times 10^{-6}$	$1\times 10^{-6}$	$9.18\times 10^{-7}$	$6\times 10^{-5}$
F9	0.47577	$3\times 10^{-7}$	$1.30\times 10^{-7}$	$1\times 10^{-6}$	$-2.46\times 10^{-6}$	$6\times 10^{-5}$

**Table 21:** Measurement results of the harmonic tests.

**Test Name**	TVE (%)	$u_{TVE}\;(-)$	FE (Hz)	$\sigma_{FE}\;(\mathrm{Hz})$	RFE (Hz/s)	$\sigma_{RFE}\;(\mathrm{Hz}/s)$
H1	0.55204	$3\times 10^{-7}$	$8.13\times 10^{-7}$	$1\times 10^{-6}$	$-1.60\times 10^{-6}$	$4\times 10^{-5}$
H2	0.76254	$5\times 10^{-7}$	$7\times 10^{-6}$	$1\times 10^{-6}$	$-1.51\times 10^{-6}$	$4\times 10^{-5}$
H3	0.89909	$4\times 10^{-7}$	$7\times 10^{-6}$	$1\times 10^{-6}$	$-1.17\times 10^{-6}$	$4\times 10^{-5}$
H4	0.82986	$5\times 10^{-7}$	$1.4\times 10^{-5}$	$1\times 10^{-6}$	$1.65\times 10^{-6}$	$4\times 10^{-5}$
H5	0.91423	$3\times 10^{-7}$	$-6\times 10^{-6}$	$1\times 10^{-6}$	$1.99\times 10^{-6}$	$4\times 10^{-5}$
H6	0.54446	$3\times 10^{-7}$	$7\times 10^{-6}$	$1\times 10^{-6}$	$2.26\times 10^{-6}$	$4\times 10^{-5}$
H7	0.72270	$3\times 10^{-7}$	$2.64\times 10^{-7}$	$1\times 10^{-6}$	$3.37\times 10^{-8}$	$4\times 10^{-5}$
H8	0.76340	$3\times 10^{-7}$	$2.86\times 10^{-9}$	$1\times 10^{-6}$	$3.14\times 10^{-7}$	$4\times 10^{-5}$
H9	0.85068	$5\times 10^{-7}$	$-1.3\times 10^{-5}$	$1\times 10^{-6}$	$-2.13\times 10^{-7}$	$5\times 10^{-5}$
H10	0.6461	$4\times 10^{-6}$	$2.0\times 10^{-5}$	$1\times 10^{-6}$	$-4.50\times 10^{-6}$	$7\times 10^{-5}$
H11	0.81305	$3\times 10^{-7}$	$9\times 10^{-6}$	$1\times 10^{-6}$	$2.50\times 10^{-6}$	$5\times 10^{-5}$
H12	0.70363	$2\times 10^{-7}$	$-2\times 10^{-6}$	$1\times 10^{-6}$	$-2.99\times 10^{-6}$	$5\times 10^{-5}$
H13	0.97941	$2\times 10^{-7}$	$-2\times 10^{-6}$	$1\times 10^{-6}$	$-1.06\times 10^{-6}$	$5\times 10^{-5}$
H14	0.8861	$9\times 10^{-7}$	$2.6\times 10^{-5}$	$1\times 10^{-6}$	$7.53\times 10^{-7}$	$6\times 10^{-5}$
H15	0.43403	$3\times 10^{-7}$	$-5.48\times 10^{-7}$	$1\times 10^{-6}$	$9.23\times 10^{-7}$	$5\times 10^{-5}$
H16	0.8436	$6\times 10^{-7}$	$1.3\times 10^{-5}$	$1\times 10^{-6}$	$-1.27\times 10^{-6}$	$6\times 10^{-5}$

**Table 22:** Measurement results of the phase tests.

**Test Name**	TVE (%)	$u_{TVE}\;(-)$	FE (Hz)	$\sigma_{FE}\;(\mathrm{Hz})$	RFE (Hz/s)	$\sigma_{RFE}\;(\mathrm{Hz}/s)$
P1	0.31161	$3\times 10^{-7}$	$-3\times 10^{-6}$	$1\times 10^{-6}$	$5\times 10^{-5}$	$4\times 10^{-5}$
P2	0.13178	$6\times 10^{-7}$	$-2\times 10^{-6}$	$1\times 10^{-6}$	$1.93\times 10^{-6}$	$4\times 10^{-5}$
P3	0.84269	$3\times 10^{-7}$	$-1\times 10^{-6}$	$1\times 10^{-6}$	$-4.54\times 10^{-6}$	$4\times 10^{-5}$
P4	0.71170	$3\times 10^{-7}$	$-9.34\times 10^{-7}$	$1\times 10^{-6}$	$-1.11\times 10^{-6}$	$4\times 10^{-5}$
P5	0.83629	$4\times 10^{-7}$	$6\times 10^{-6}$	$1\times 10^{-6}$	$-1\times 10^{-5}$	$4\times 10^{-5}$
P6	0.08218	$9\times 10^{-7}$	$-8.40\times 10^{-8}$	$1\times 10^{-6}$	$-9.72\times 10^{-6}$	$4\times 10^{-5}$
P7	0.76181	$3\times 10^{-7}$	$-8.71\times 10^{-7}$	$1\times 10^{-6}$	$-3.91\times 10^{-6}$	$4\times 10^{-5}$
P8	0.2903	$1\times 10^{-6}$	$1.1\times 10^{-5}$	$1\times 10^{-6}$	$-5.17\times 10^{-6}$	$4\times 10^{-5}$
P9	0.72415	$3\times 10^{-7}$	$-2\times 10^{-6}$	$1\times 10^{-6}$	$-7.36\times 10^{-6}$	$4\times 10^{-5}$
P10	0.53129	$5\times 10^{-7}$	$-1.5\times 10^{-5}$	$1\times 10^{-6}$	$-5.18\times 10^{-6}$	$4\times 10^{-5}$
P11	0.89278	$3\times 10^{-7}$	$-2\times 10^{-6}$	$1\times 10^{-6}$	$-9.10\times 10^{-6}$	$4\times 10^{-5}$

## Data Availability

Not applicable.
